# The effect of lying on memory in daily life: Does motivation matter?

**DOI:** 10.1002/pchj.709

**Published:** 2023-12-17

**Authors:** Yan Li, Zhiwei Liu

**Affiliations:** ^1^ School of Education and Psychological Science Sichuan University of Science and Engineering Zigong China

**Keywords:** daily life, lying, memory, motivation

## Abstract

Recently, there has been renewed interest in the effect of lying on memory. A growing body of studies has documented that lying can impair memories and cause memory disruptions, such as forgetting and false memories, to a greater degree than telling the truth. This study aimed to investigate whether motivation plays a role in the effect of lying on memory. The present study utilized a daily life paradigm and manipulated three conditions: truth telling, internally motivated lying, and externally motivated lying. We asked participants to engage in a shopping task and to tell lies (externally motivated lying group) or to choose between telling the truth (truth‐telling group) and telling lies (internally motivated lying group) in the interview. Forty‐eight hours later, the participants were instructed to truthfully carry out multiple memory assessments. The principal findings of this research are that lying can result in memory impairments, and internally motivated lying can lead to greater impairment in source memory than externally motivated lying. Moreover, no significant differences between the two lying groups were found in the memory tests. The empirical findings of this study provide new insights into the effect of lying on memory.

## INTRODUCTION

Telling lies is a common behavior, and people do it every day (Riesthuis et al., 2021). Deception or lying has been defined as deliberate behaviors meant to mislead people to gain benefits or avoid losses (Abe, 2009). Liars might obtain what they want as a result of their lies, but these lies may also result in memory impairment (for a review see Battista & Otgaar, 2022). Memory can be contaminated by the telling of lies and may result in further forgetting and/or more false memories (Otgaar & Baker, 2018).

Several types of lies have been studied, and researchers have found that memory impairment varies with the types of lies (e.g., false denial, feigning amnesia, and fabricating). A denial‐induced forgetting (DIF) effect has been observed when people use a false denial strategy (denying details or events that happened; Battista, Curci, et al., 2021; Otgaar et al., 2016; Otgaar & Baker, 2018). Specifically, liars who falsely deny the details of a video or the items asked in an interview make more omission errors (omission errors are forgetting errors, and commission errors are false memories) than honest people (Battista et al., 2020; Battista, Curci, et al., 2021; Otgaar et al., 2020). Previous studies have indicated that feigning amnesia, which involves claiming memory loss or the inability to recall specific details or events, can disrupt memories and cause memories of the details or events to become weaker (e.g., Mangiulli, van Oorsouw, et al., 2019; Romeo, Otgaar, Smeets, Landstrom, & Boerboom, 2019; van Oorsouw & Giesbrecht, 2008). After viewing or executing a mock crime, those who initially claimed amnesia remembered fewer correct details and made more errors than those who confessed (Mangiulli et al., 2018, 2022). Fabricating completely false details or events could result in more false memories, and fabricators often misremember the details or events that they have confabulated (e.g., Battista, Mangiulli, et al., 2021; Pezdek et al., 2009; Riesthuis et al., 2020). Some studies also found that immediately providing confirmatory feedback to liars' confabulated responses can increase their false memories (Frost et al., 2003; Hanba & Zaragoza, 2007; Zaragoza et al., 2001).

The typical procedure recently used to study lying and memory often contains two sessions (Battista, Mangiulli, et al., 2021; Bücken et al., 2022; Li et al., 2022b). During the initial session, the participants are either shown a video or instructed to simulate the act of committing a mock crime. Subsequently, the participants are asked to complete a baseline memory test and give honest answers to questions about the video or mock crime. Then, the participants are randomly assigned to the honest or lying groups and asked to provide responses in an interview in which several questions are asked about the stimulus or mock crime. During the interview, the group of honest participants is instructed to provide truthful responses, and the lying group is asked to provide deceptive responses. The required deceptive responses depend on the specific type of lying that the study is focused on. The lying group is asked to respond to the questions with denials (false denial), or to feign memory loss and claim not to remember anything (feigning amnesia), or to provide completely fabricated responses (fabricating) in the interview. After an interval (often 1 or 2 days), the second session is held, and all participants are asked to respond honestly in a final memory test concerning the stimulus or the mock crime and the interview from the first session. Error rates and other measures (such as belief and memory ratings) of the honest and lying groups are compared to determine the effects of lying on memory. Typically, the final memory test reveals that the lying group forgets more memories and/or has more false memories than the honest group (e.g., Ackil & Zaragoza, 2011; Battista, Otgaar, et al., 2021; Mangiulli et al., 2020; van Oorsouw & Merckelbach, 2004; Vieira & Lane, 2013).

The memory and deception (MAD) framework (Otgaar & Baker, 2018) is frequently used to explain the effects of lying on memory (e.g., Battista, Mangiulli, et al., 2021; Battista et al., 2020; Battista & Otgaar, 2022; Mangiulli et al., 2020; Otgaar et al., 2018, 2020; Romeo, Otgaar, Smeets, Landström, & Jelicic, 2019). In their work, Otgaar and Baker (2018) suggested that cognitive resource consumption varies with deceptive strategy. A small cognitive effort might be required when people falsely deny something (Otgaar & Baker, 2018). More cognitive resources are consumed when liars feign amnesia than when they engage in false denial (Otgaar & Baker, 2018). Furthermore, liars who fabricate completely false details may need to exert much greater cognitive effort than those who employ the other two deceptive strategies (Otgaar & Baker, 2018). In short, the memory outcomes due to deception are justified by the different cognitive loads employed to lie in different lie situations (Otgaar & Baker, 2018).

Few studies, however, have considered motivation when the effects of lying on memory are examined. It has been suggested that people tell lies out of various motivations (Elaad & Gonen‐Gal, 2022; McArthur et al., 2022; Sneddon, 2021). We defined two types of motivated lying. Internally motivated lying is driven by the liar's own desires to avoid personal loss or punishment, or to gain invisible benefits such as reputation, or to achieve prosocial outcomes. Internally motivated lies also can be told for nothing, or just for amusement, or owing to a lack of moral values. Externally motivated lying is driven by external requirements, pressures, threats, or (in)visible temptations (such as money and sex), and externally motivated lies are told owing to external circumstances inducing the lying behavior. In the study conducted by Li et al. (2022b), participants were presented with a choice between telling the truth or lying, and they were grouped based on the choice they made. The participants who voluntarily chose to engage in lying could receive additional benefits for their participation in the investigation, and the researchers found that liars exhibited higher error rates in both the source and destination memory tests compared with the truth‐telling group (Li et al., 2022b). Riesthuis and his colleagues examined the effects of self‐generated deceptive behavior on memory (Riesthuis, Otgaar, et al., 2022). Two conditions were manipulated in their study. The participants in the strong incentive to cheat condition told lies to avoid financial punishment, and those in the weak incentive to cheat condition could choose to lie to achieve a prosocial outcome. Riesthuis, Otgaar, et al. (2022) found that liars, regardless of their motives, made more memory errors than honest participants. Moreover, they also observed a lack of significant differences between the strong and weak incentive to cheat conditions. Romeo and his colleagues asked participants who had just viewed a traumatic virtual reality video of an airplane crash to make a choice between truth‐telling and falsely denying in the interview (Romeo, Otgaar, Smeets, Landstrom, & Boerboom, 2019). However, half of the participants who chose to tell the truth were instructed to falsely deny, as the number of participants who chose to falsely deny was too small. Romeo and his colleagues (Romeo, Otgaar, Smeets, Landstrom, & Boerboom, 2019) found that the participants who were motivated to tell the truth but were instructed to falsely deny made more memory errors than those who were simply motivated to tell the truth. Obviously, in previous studies, Riesthuis, Otgaar, et al. (2022) examined the effects of internally motivated lying on memory, while Romeo, Otgaar, Smeets, Landström, and Jelicic (2019) and Li et al. (2022b) investigated the effects of externally motivated lying on memory. To the best of our knowledge, no previous study has provided information on the differences between the effects of internally and externally motivated lying on memory.

To respond to the call from Battista and Otgaar (2022) for more research in the field and to develop a better understanding of the effects of lying on memory, this exploratory study was designed to determine whether motivation plays a role in this effect. It is important to examine this issue to ascertain whether it is necessary to take motivation as a variable when investigating the effects of lying on memory. In some legal situations as well as in daily life, people tell lies for a variety of reasons. Some criminals may lie for their own benefit, such as evading punishment, and some witnesses may give deceptive responses in police investigations or court inquiries as a result of external threats. Moreover, people inflate the price of their car in order to appear more respectable, and children exaggerate their academic performance in order to obtain material rewards from their parents. Thus, people may tell lies for either internal or external motivations. The present study aimed to investigate the effects of both internally and externally motivated lying on memories and the differences between them.

In this study, a daily life scenario and a simulated shopping task were employed for this purpose. The participants were instructed to visit a small store and acquire 10 items without making any payment. In an interview, the participants were queried about their shopping lists. Two female interviewers were invited to conduct the interview to test destination memory. Some participants were asked to lie in the interview, while the remaining participants were provided with choices between telling lies or telling the truth. The participants who chose to tell lies were categorized as in the internally motivated lying group, and those instructed to tell lies were categorized as in the externally motivated lying group. All participants were requested to return after 2 days to honestly complete the final memory test, which consisted of an item memory test, source memory test, and destination memory test. Given that memory and emotion are interrelated (Bisby et al., 2016; Phelps, 2004) and that memory might be influenced by working memory (Gerrie & Garry, 2007; Peters et al., 2007), the current study evaluated both emotion and working memory. Based on previous studies (Li et al., 2022a, 2022b; Li & Liu, 2021), we hypothesized that emotion and working memory would play no role in the present study.

To further explore and validate the effects of deception on beliefs and memories (Battista, Mangiulli, et al., 2021; Li et al., 2022a), the present study assessed the beliefs and memories of each test entry and hypothesized that they could be affected by deceptive behavior. To evaluate the impact of internally and externally motivated lying on memory, the differences in memory test results among the groups were compared. Based on previous studies that demonstrated that lying impairs memory (see the review by Battista & Otgaar, 2022), we hypothesized that lying, whether internally or externally motivated, may lead to more memory errors (including forgetting and commission errors) than telling the truth and cause lying effects. It has been suggested that there are no significant differences in the cognitive signatures of lying between liars who are instructed to lie and those who self‐initiate lying (Geven et al., 2020). Thus, we also hypothesize that there are no performance differences in memory tests between liars with these motivations.

Moreover, people across cultures usually accuse each other of lying because of lying's immoral nature (Shen et al., 2016). This may make deceptive behaviors become unwelcome memories, such that liars tend to forget them intentionally. It has been argued that people tend to forget their unwelcome memories, which causes their memories of relevant acts to become more impoverished (Anderson & Hanslmayr, 2014). Intentional forgetting can not only impair the encoding stage of memory but also impoverish the memory trace within long‐term memory (Fawcett et al., 2016). The internally motivated lying group chose to tell lies to achieve a strategic purpose (more money), which made their deceptive behaviors seem more immoral than those of the externally motivated lying group. As an exploratory investigation, we examined if the effect size of lying on memory (the differences in memory performance between the lying groups and the honest group) differed by motivation.

## METHODS

### Participants

We conducted an a priori power analysis using G*Power software (Faul et al., 2007; version 3.1.9.7) to determine the required sample size for detecting significant effects. For this power analysis, we specified the use of an *F*‐test one‐way analysis of variance (ANOVA), an effect size *f* of 0.3, a power of 0.8, and an alpha level of 0.05. A medium effect size *f* of 0.3 was chosen based on previous deception studies using similar experimental manipulations and memory measures that yielded effects in this range (e.g., Geven et al., 2020; Polage, 2019). A power of 0.8 represents a conventional trade‐off between having adequate statistical power and minimizing research costs and participant burdens, as discussed by Cohen (1992) and in the guidebook by Ellis (2010). The power analysis indicated a minimum required sample size of 111 participants. A total of 120 volunteers (64 females) aged 21–26 years (*M* = 21.21 years, *SD* = 2.07) were recruited. Four participants failed to take part in session two for personal reasons. Therefore, a total sample of 116 participants completed this study. All participants gave their written informed consent as per the Helsinki Declaration. All of the participants were paid for their participation. This study was approved by the Ethics Committee of the School of Education and Psychological Science at Sichuan University of Science and Engineering.

### Design

The study adopted a single‐variable between‐subject design. After completing the shopping task, 40 participants (including two who did not attend session 2) were asked to tell lies (the externally lying group), and the others were allowed to choose between lying and telling the truth in the interview. Thirty‐eight participants chose to tell the truth (the honest group), and 42 participants chose to tell lies (the internally motivated lying group), and one participant from each group failed to join session 2. Therefore, the participants were divided into three groups (the honest group, the internally motivated lying group, and the externally motivated lying group), and group membership was taken as the independent variable. In addition, the participants took several memory tests, and the error rates and ratings on the memory tests served as the dependent variables.

### Procedure

Following previous studies (Li et al., 2022a, 2022b; Li & Liu, 2021), a daily life paradigm that contained two sessions was used in the present study.

#### Session 1

A compact shop was established on the 14th floor of the research institution. A total of 20 varieties of products were made available for purchase, namely chewing gum, pie, cookies, seaweed, bread, coca‐cola, chocolate, soap, toothbrushes, tissues, toothpaste, towels, hangers, noodles, garbage bags, coffee, N95 masks, shampoo, water, and swabs.

The participants engaged in individual participation and were initially instructed to simulate a shopping trip in the compact store, where they were required to purchase 10 different products without time limitation. Subsequently, the participants were instructed to utilize their smartphones to scan a payment QR code, simulating a transaction process that did not involve real currency, thus completing the mock payment. Following the shopping activity, the participants were presented with a distractor task, involving playing Tetris, for a duration of 5 min. Then, the participants took part in a baseline memory test, where they were instructed to recall and write down the products they had purchased. During the test, the participants were also required to provide ratings for their belief and memory regarding each item they purchased. They were asked to rate their belief strength on a scale of 1 to 8, with 1 representing no belief and 8 indicating a strong belief. Similarly, they were asked to rate their memory of purchasing each item on a scale of 1 to 8, with 1 representing no memory at all and 8 indicating a clear and complete memory. Upon completion of the baseline memory test, the participants were once again presented with a distractor task, involving playing Tetris, for a duration of 5 min.

The participants were informed that they would be interviewed by two interviewers on the 13th floor who were unaware of the items they had purchased. These interviewers would pose questions regarding their shopping lists. In the externally motivated lying group, participants were specifically instructed to provide deceptive or false responses during the interview. The other participants were told that they could choose to answer all of the questions honestly (the honest group) or deceptively (the internally motivated lying group). Based on a previous study (Li et al., 2022b), the participants were additionally told that they would obtain more cash (6 yuan) for their participation if they chose to lie in the interview. During the interviews, the participants who chose to tell the truth were asked to respond honestly to each question, and those who chose to tell lies were asked to provide deceptive answers.

Subsequently, the participants were escorted to an interview room, where they were instructed to provide responses as accurately as possible according to the instructions provided for each group. To test destination memory, two females who were completely unfamiliar to all of the participants were invited as the interviewers. The shopping lists were made available to the interviewers while the participants were undergoing the baseline memory test. A total of 10 questions were prepared for the interview, focusing on five randomly selected items from the shopping lists and five items that were not available for sale in the small store. One of the interviewers posed questions specifically related to the items from the shopping lists, while the other interviewer inquired about the remaining items that were not part of the participants' purchases. Following a fixed question structure of “Did you buy X?”, the interviewers took turns to ask questions, maintaining consistency throughout the interview process. In answering the questions, the participants in the truth‐telling group were directed to give positive responses to the questions about the items from their shopping lists and give negative responses to the questions about the items that were not sold in the small store. In contrast, the participants in the internally and externally motivated lying groups were asked to give negative responses to the questions about the items from their shopping lists (false denial) and give positive responses to the questions about the items not sold in the small store (fabricating).

Following a previous study (Li et al., 2022b), we also planned to clarify the relationships among lying, emotion, working memory capability, and memory in the present study. The participants were asked to report how nervous they felt during the interview by using a 10‐point Likert scale (1 = *not nervous at all*, 10 = *very nervous*), and their working memory was assessed with the Wechsler Adult Intelligence Scale‐III Digit Span subtest (Wechsler, 1997). In the subtest, the participants were asked to repeat 3–11 digits both forward and backward. The number of digits the participants could repeat both forward and backward was used as their score on the subtest.

#### Session 2

Two days later, all participants were asked to return to the laboratory and engage in a final memory test. In the final memory test, all participants, regardless of the group assignment, were instructed to respond honestly. The test consisted of three subtests: the item memory test, the source memory test, and the destination memory test. These subtests aimed to assess different aspects of participants' memory performance. During the item memory test, the participants were prompted to freely recall their shopping lists and provide belief and memory ratings for each item. They were asked to rate their belief strength on a scale of 1 to 8, with 1 indicating no belief and 8 indicating a strong belief. Similarly, they were asked to rate their memory of purchasing each item on a scale of 1 to 8, with 1 representing no memory at all and 8 indicating a clear and complete memory. In the source memory test, participants were presented with a total of 20 items. This set included 10 items that were part of their shopping lists (with five of them being specifically asked about during the interview), five items that were asked about during the interview but that were not available for purchase in the small store, and another five items that were neither asked about during the interview nor sold in the small store.

During the source memory test, participants were instructed to determine whether specific items had been asked about during the interview. They were then asked to provide belief ratings, indicating the strength of their belief regarding whether they were or were not asked about each item during the interview, on a scale of 1 to 8. A rating of 1 represented no belief, while a rating of 8 indicated a strong belief. Furthermore, participants were required to provide memory ratings, indicating their actual recollection of whether they were or were not asked about each item during the interview, on a scale of 1 to 8. A rating of 1 signified no memory at all, whereas a rating of 8 indicated a clear and complete memory.

During the destination memory test, participants were presented with the items that were specifically asked about during the interview, as well as photographs of the interviewers. They were then asked to recall and indicate which interviewer had asked them about particular items during the interview by pointing out the corresponding interviewer's photograph. Additionally, participants were asked to provide belief ratings, indicating the strength of their belief that a specific interviewer was the one who asked them about each item during the interview. The belief ratings ranged from 1 (no belief) to 8 (strong belief). Moreover, participants were also requested to provide memory ratings, indicating their recollection of whether a particular interviewer was the one who asked them about each item during the interview. The memory ratings ranged from 1 (no memory) to 8 (clear and complete memory). After the final memory tests, the study's purpose was explained to the participants, and we stated that we do not encourage lying.

### Scoring

The baseline memory test, the item memory test, the source memory test, and the destination memory test were rated by the memory scores. Memory scoring was performed by the first author, and a student assistant who was completely blinded to the experimental design double checked the scores to ensure accuracy.

To calculate the number of correctly reported details, a point was given for each correct answer in each memory test. No answer or a completely wrong answer was given a score of zero. The maximum score obtainable was 10 in the baseline memory test, the item memory test, and the destination memory test, whereas the maximum score obtainable was 20 in the source memory test. The memory test error rates for each participant were calculated as follows: the obtained score was subtracted from the maximum score, and the resulting sum was then divided by the maximum score. The average belief and memory ratings for each participant were calculated as follows: the belief ratings and memory ratings of the items that obtained a score were summed, and the result was then divided by the number of items that obtained a score. Thus, the dependent variable error rates are equal to the sum of the error rates of all participants divided by the number of participants, and the average belief and memory ratings are equal to the sum of the belief and memory ratings of all participants divided by the number of participants.

## RESULTS

Following previous studies (Li et al., 2022a, 2022b; Li & Liu, 2021), all data were analyzed in the *R* system with the lme 4 package. A linear mixed‐effects (LMEs) model was used to analyze the belief and memory ratings, and a generalized linear mixed‐effects (GLMEs) model was used to analyze error rates, with participants and items serving as crossed random effects (Baayen et al., 2008). Moreover, *z* or *t* values of larger than 1.96 were considered statistically significant, and larger *z* or *t* values were considered to indicate greater effects, following previous studies (Baayen et al., 2008; Zang et al., 2021). The descriptive and inferential statistics are shown in Tables 1 and 2, respectively.

**TABLE 1:** Mean error rates and ratings in the memory tests for each condition.

Memory test	Group	Error rate (%)	Memory rating	Belief rating
Baseline memory test	Truth‐telling	10.03 (1.57)	6.98 (0.09)	7.67 (0.05)
	Internally motivated lying	9.02 (1.42)	7.08 (0.06)	7.69 (0.03)
	Externally motivated lying	10.05 (1.57)	6.86 (0.09)	7.73 (0.05)
Item memory test	Truth‐telling	10.33 (1.59)	6.35 (0.09)	7.42 (0.06)
	Internally motivated lying	10.29 (1.51)	6.45 (0.07)	7.54 (0.05)
	Externally motivated lying	10.59 (1.61)	6.26 (0.11)	7.43 (0.08)
Source memory test	Truth‐telling	21.54 (1.51)	5.63 (0.09)	6.15 (0.08)
	Internally motivated lying	29.57 (1.61)	5.67 (0.08)	5.75 (0.08)
	Externally motivated lying	27.29 (1.66)	5.77 (0.09)	5.99 (0.09)
Item category 1	Truth‐telling	14.13 (2.58)	5.89 (0.13)	6.34 (0.15)
	Internally motivated lying	16.58 (2.64)	6.25 (0.13)	6.34 (0.14)
	Externally motivated lying	14.21 (2.63)	6.21 (0.14)	6.39 (0.14)
Item category 2	Truth‐telling	28.81 (3.35)	4.59 (0.19)	5.27 (0.17)
	Internally motivated lying	54.15 (3.49)	4.83 (0.21)	4.69 (0.21)
	Externally motivated lying	48.37 (3.69)	4.81 (0.21)	4.76 (0.23)
Item category 3	Truth‐telling	29.19 (3.35)	5.93 (0.17)	6.43 (0.16)
	Internally motivated lying	30.61 (3.31)	5.79 (0.17)	6.22 (0.15)
	Externally motivated lying	29.38 (3.43)	5.99 (0.17)	6.46 (0.16)
Item category 4	Truth‐telling	14.05 (2.56)	5.98 (0.17)	6.47 (0.14)
	Internally motivated lying	16.59 (2.61)	5.46 (0.17)	5.67 (0.15)
	Externally motivated lying	16.76 (2.75)	5.73 (0.18)	5.96 (0.19)
Destination memory test	Truth‐telling	14.91 (1.86)	5.04 (0.12)	5.94 (0.13)
	Internally motivated lying	42.03 (2.49)	4.50 (0.13)	4.93 (0.14)
	Externally motivated lying	43.22 (2.64)	4.68 (0.15)	4.91 (0.17)

*Note*: The standard errors of means are shown in parentheses. Item categories 1, 2, 3, and 4 are four kinds of items in the source memory test. Item category 1 refers to the items that the participants bought and were asked about in the interview; item category 2 refers to the items that were on the shopping lists but not asked about in the interview; item category 3 refers to the items that were not on the shopping lists but were asked about in the interview; and item category 4 refers to the items that were not sold in the small store and were not asked about in the interview.

**TABLE 2:** Statistical effect for the memory tests.

		Error rate			Memory			Belief		
		*b*	*SE*	*z*	*b*	*SE*	*t*	*b*	*SE*	*t*
Baseline memory test	1 versus 2	0.11	0.25	0.44	0.1	0.18	0.57	0.03	0.09	0.29
	1 versus 3	0.01	0.25	0.04	0.12	0.24	0.51	0.06	0.09	0.59
	2 versus 3	0.17	25	0.66	0.21	0.19	1.12	0.04	0.09	0.41
Item memory test	1 versus 2	0.02	0.26	0.1	0.07	0.28	0.24	0.13	0.13	1.02
	1 versus 3	0.04	0.25	0.16	0.15	0.33	0.46	0.01	0.16	0.03
	2 versus 3	0.09	0.31	0.32	0.22	0.26	0.82	0.13	0.15	0.88
Source memory test	1 versus 2	0.44	0.13	**3.38**	0.03	0.28	0.11	0.39	0.24	1.66
	1 versus 3	0.31	0.14	**2.17**	0.19	0.28	0.67	0.09	0.22	0.43
	2 versus 3	0.14	0.12	1.19	0.17	0.27	0.61	0.29	0.23	1.27
Item category 1	1 versus 2	0.25	0.36	0.69	0.22	0.27	0.81	0.12	0.28	0.43
	1 versus 3	0.01	0.35	0.001	0.32	0.25	1.27	0.08	0.25	0.33
	2 versus 3	0.19	0.32	0.62	0.11	0.28	0.39	0.19	0.26	0.72
Item category 2	1 versus 2	1.63	0.42	**3.84**	0.04	0.41	0.11	0.69	0.38	1.81
	1 versus 3	1.06	0.36	**2.98**	0.25	0.43	0.59	0.43	0.41	1.05
	2 versus 3	0.35	0.28	1.23	0.33	0.44	0.75	0.33	0.45	0.73
Item category 3	1 versus 2	0.08	0.26	0.29	0.13	0.34	0.37	0.21	0.29	0.71
	1 versus 3	0.01	0.14	0.05	0.06	0.36	0.18	0.01	0.29	0.03
	2 versus 3	0.09	0.29	0.31	0.21	0.31	0.65	0.18	0.27	0.66
Item category 4	1 versus 2	0.2	0.49	0.41	0.29	0.43	0.69	0.64	0.37	1.47
	1 versus 3	0.26	0.49	0.53	0.02	0.45	0.04	0.28	0.39	0.72
	2 versus 3	0.07	0.53	0.14	0.32	0.42	0.74	0.37	0.39	0.97
Destination memory	1 versus 2	1.92	0.35	**5.45**	0.61	0.35	1.74	1.12	0.41	**2.77**
	1 versus 3	2.05	0.39	**5.26**	0.47	0.36	1.33	1.18	0.44	**2.72**
	2 versus 3	0.03	0.23	0.15	0.13	0.35	0.38	0.07	0.42	0.16

*Note*: Bold numbers indicate significant values. The numbers 1, 2, and 3 in the second column represent truth‐telling, internally motivated lying, and externally motivated lying conditions, respectively.

The shopping rates, the baseline memory test, and the interview scores were analyzed as manipulation checks and to check possible differences in memory participants. These variables are not our main dependent variables in the present study.

### Shopping

The shopping time of the participants in the mock shopping task was recorded, but no time limit was imposed. No significant differences in shopping time were observed among the groups (honest group: *M* = 121 s, *SD* = 25.3; internally motivated lying group: *M* = 117 s, *SD* = 23.9; externally motivated lying group: *M* = 130 s, *SD* = 30.1; *F* = 2.29, *p* = .11).

### Baseline memory test

Differences observed between the error rates or ratings of the different groups in the baseline memory test did not reach a significant level, as indicated in Tables 1 and 2. The findings from the baseline memory test indicated that participants in all three groups demonstrated good memory recall of their shopping lists upon completing their shopping tasks.

### Interviews

During the interviews, certain participants provided incorrect responses. For instance, individuals in the truth‐telling group sometimes gave negative responses when asked about items on their shopping lists, while liars occasionally provided negative responses regarding items that were not available for purchase in the small store. We accounted for the incorrect responses in each group (truth‐telling: 1, internally motivated lying: 15, and externally motivated lying: 16) and found a significant difference in the number of incorrect responses among the groups (*χ* ^2^ = 12.76, *p* = .005). For subsequent memory tests, the items that received incorrect responses were excluded from the data analysis.

After the interview, the participants were asked to self‐report their nervousness during the interview, and no significant differences were observed between the groups in terms of nervousness (truth‐telling: *M* = 4.43, *SD* = 2.26; internally motivated lying: *M* = 4.61, *SD* = 2.17; externally motivated lying: *M* = 4.74, *SD* = 1.74; *F* = 0.21, *p* = .81). Working memory capacity was also measured, and the difference detected among the groups was nonsignificant (truth‐telling: *M* = 15.05, *SD* = 2.12; internally motivated lying: *M* = 15.68, *SD* = 2.69; externally motivated lying: *M* = 16.08, *SD* = 2.48; *F* = 1.91, *p* = .14).

### Item memory test

As shown in Tables 1 and 2, no significant between‐group differences were identified in the item memory test, suggesting that neither internally nor externally motivated lying impaired the participants' memories of their shopping lists.

### Source memory test

As shown in Tables 1 and 2, the source memory test revealed significant differences in error rates between the truth‐telling group and the lying groups. These findings suggest that the lying groups exhibited more memory impairments compared with the truth‐telling group. Moreover, the differences between the lying groups did not reach a significant level. Other differences observed in the source memory test were not significant.

As shown in Tables 1 and 2, we divided the items in the source memory test into four categories according to whether they were on the shopping lists and whether participants had been asked about the item in the interview, as follows: items that were included on the shopping lists and subsequently queried during the interview (item category 1), items that were present on the shopping lists but were not specifically addressed or inquired about during the interview (item category 2), items that were not available for purchase in the small store but were still queried or questioned during the interview (item category 3), and items that were not available for purchase in the small store and were not mentioned or questioned during the interview (item category 4). We then compared the performances of each group in the four categories. Table 2 illustrates significant differences in error rates between the lying and truth‐telling groups specifically for item category 2. The internally and externally motivated lying groups exhibited a higher number of incorrect responses compared with the truth‐telling group specifically in item category 2. This suggests that the lying groups mistakenly believed they were being asked about the items present on their shopping lists. No significant differences were observed across item categories when comparing the groups, indicating that no other variations reached a statistically significant level.

It is noteworthy that the effect of lying on memory was greater in the internally motivated lying group than in the externally motivated lying group in the source memory test and in item category 2 (see the *z* values in Table 2).

### Destination memory test

As shown in Tables 1 and 2, the analysis revealed significant differences between the lying groups and the truth‐telling group, indicating that the lying groups exhibited a higher tendency to form false memories in recalling their destination memory compared with the truth‐telling group. In addition to the correct responses in the destination memory test, we also observed significant differences in the belief ratings between the truth‐telling group and the lying groups. The lower belief ratings in the lying groups, compared with the truth‐telling group, indicate that engaging in lying could potentially undermine their belief in destination memory. No other significant differences were observed.

## DISCUSSION

The primary objective of the present study was to examine the impact of lying on memory across three distinct conditions: truth‐telling, internally motivated lying, and externally motivated lying. As indicated in the results section, the results achieved on the final memory test did not consider items erroneously replied to during the interview. We found that lying undermined both source and destination memory, which is consistent with previous studies (Li et al., 2022a, 2022b; Li & Liu, 2021). Moreover, we did not observe any significant differences between the internally and externally motivated lying groups. A more specific discussion of each finding is given below.

In the interviews, we observed that liars responded incorrectly more often than honest participants. The significant differences in incorrect responses between the lying and truth‐telling groups suggest that it was more difficult for the respondents to tell lies than to tell the truth. It has been suggested that more cognitive effort may be required to inhibit automatically activated truthful responses and then to construct lies (Walczyk et al., 2014). Therefore, the higher number of incorrect responses among the liars might result from a failure to inhibit truthful responses.

In the present study, our focus was on investigating how engaging in lying influences the accuracy and reliability of source memory. The participants in the lying groups exhibited higher error rates than those in the truth‐telling group, suggesting that lying may have impaired the source memory for the interview. The obtained result aligns with previous research findings (Battista, Mangiulli, et al., 2021; Li et al., 2022a) and supports the notion that individuals who engage in lying encounter challenges in accurately remembering the details of their lies. Additionally, a significant difference was observed between the lying and truth‐telling groups concerning the items on the shopping lists that were not specifically addressed in the interview. This finding suggests that engaging in lying was associated with a higher likelihood of forming false memories. This finding aligns with previous studies (Li & Liu, 2021; Riesthuis, Mangiulli, et al., 2022) and supports the notion that lying can lead to increased memory impairments and can have a detrimental effect on memory performance.

In this study, we also discovered effects of lying on destination memory. Destination memories refer to recollection of the recipients of previously disclosed information (Marsh & Hicks, 2002). The liars need to remember to whom they lied if they want to avoid exposing their lies. Consistent with previous research (Li et al., 2022a, 2022b, Li & Liu, 2021), the present study finds that the lying groups displayed higher error rates in the destination memory test compared with the truth‐telling group. As a result, the lying groups forgot more and formed more false memories about to whom they lied regarding specific items. Moreover, the liars also reported lower belief ratings than the truth tellers in the destination memory test, suggesting that liars exhibited higher levels of uncertainty about what they had told to whom regarding particular items than did the truth tellers. The results of the source and destination memory tests show that the lying groups had more false memories regarding what and to whom they had lied. Therefore, lying can have extensive effects on memory, increasing the risk of lies being exposed.

The effects of lying on memory observed in the present study can potentially be understood through the MAD framework proposed by Otgaar and Baker (2018). According to the MAD framework, lying places greater demands on cognitive resources than truth telling. Liars have to suppress the honest response activated in memory and construct a deceptive response instead. This effortful process taxes cognitive resources that could otherwise be utilized for encoding details of the social context into memory. The truth is activated by social context, and truthful responses may be automatically activated and thus need to be inhibited to formulate deceptive responses when one decides to lie (Walczyk et al., 2014). Thus, it might take more time to provide a deceptive response than to tell the truth (Suchotzki et al., 2017). It has been suggested that lying or deception may result in a greater cognitive load than telling the truth, and that more cognitive effort is needed for liars to tell lies (Otgaar & Baker, 2018). In the current study, participants in the lying conditions had to exert cognitive control to inhibit truthful responses and construct lies during the interview. This extra exertion potentially reduced their ability to commit contextual details, such as which items were asked about by which interviewer, to memory. This cognitive process of inhibiting the truth and generating deceptive responses could have placed additional demands on their working memory capacities. Indeed, telling lies typically requires more cognitive effort and consumes additional cognitive resources compared with telling the truth; thus, liars have fewer cognitive resources with which to encode or monitor details, such as the items they lied about or the person they lied to. In contrast, participants in the truth‐telling group did not face the need to inhibit or suppress the automatically activated truth when responding to interview questions. Because the truth tellers were instructed to provide honest and truthful responses, they could freely rely on their natural memory retrieval processes. Without the extra cognitive taxation from lie construction, truth tellers likely had greater cognitive resources available to encode the social context, enabling better memory for interview details. Therefore, the truth tellers likely had more cognitive resources than those in the lying groups to encode and monitor the social context and thus retain more accurate memories concerning who asked about what items in the interview.

No significant differences in the memory tests between the internally and externally motivated lying groups were observed, suggesting that deceptive motivation did not modulate the liars' performances on the memory tests. However, we found that the effects of lying on memory were greater under the internally motivated lying condition than under the externally motivated lying condition on the source memory tests (see the *z* values in Table 2). There are several possible explanations for this result. First, it has been suggested that deception may rely primarily on working memory (Christ et al., 2009), and working memory may be affected by motivation (Krawczyk & D'Esposito, 2013). It has been demonstrated that motivation can amplify enhancement and suppression in the frontal regions involved in working memory when participants are presented with trials with the highest reward compared with when they are presented with trials using a nonrewarded stimulus (Krawczyk et al., 2007). Financial incentives may increase motivation in the working memory task and require correspondingly greater neural effort for encoding (Taylor et al., 2004). In this study, the internally motivated lying group chose to tell lies for greater benefit, and the externally motivated lying group was instructed to tell lies. Thus, we may say that the internally motivated lying group was more motivated and had a stronger incentive to tell lies than the externally motivated lying group. High motivation and strong incentives may have affected the working memory for the internally motivated lying group, resulting in a greater effect of lying on memory.

Another possible explanation for the greater effect of internally motivated lying might involve individual differences. It has been reported that the tendency to lie is associated with personal characteristics such as personality and morality (Palena et al., 2022). The participants in the internally motivated lying and truth‐telling groups chose to tell lies or the truth in the interview. Thus, we may say the two groups might have large individual differences in their lying tendencies. On the other hand, the externally motivated lying and truth‐telling groups might share some similarities in their personal characteristics because the former group was randomly assigned. Individual differences might be another reason for the difference in the effect size of lying on memory between the internally and externally motivated lying groups.

The internally motivated lying group chose to lie to gain greater benefit, and the externally motivated lying group was instructed to lie in the present study. Thus, we may say that there was a difference in motivation to lie between the internally and externally motivated groups. However, our findings showed no significant performance differences on memory tests between the internally and externally motivated lying groups, consistent with a previous study by Riesthuis, Otgaar, et al. (2022) that reported no significant differences in memory tests between the strong and weak incentive to cheat conditions. In their study, Riesthuis, Otgaar, et al. (2022) asked participants in the “strong incentive to cheat” condition to tell lies to avoid a financial penalty, while those in the “weak incentive to cheat” condition were given prosocial reasons for their lies. Thus, combining the findings from the present study with those of Riesthuis, Otgaar, et al. (2022), we may conclude that internally motivated lying might not lead to more memory loss than other types of motivated lying. Moreover, both the present study and that of Riesthuis, Otgaar, et al. (2022) identified the effects of lying on memory. Deception or lying is defined as “a psychological process by which one individual deliberately attempts to convince another person to accept as true what the liar knows to be false …to gain some type of benefit or to avoid loss” (Abe, 2009, p. 594). The results from the present study and Riesthuis, Otgaar, et al. (2022) indicate that the effect of lying on memory can be observed regardless of why liars tell lies.

The present study adds to the growing body of research indicating that lying impairs memory. The results are useful in expanding our understanding of the effects of lying on memory. This study provides empirical evidence that lying motivation does not modulate the occurrence of the effects of lying on memory but rather the effect size of lying on memory. Moreover, the findings of the present study also suggest that randomly assigning participants to the experimental conditions does not contaminate the reliability of observations in the field. The findings may also have important practical implications for the use of testimony in the legal system. The findings that lying impairs memory, particularly for contextual source details, suggest that witnesses or suspects who have lied may have greater difficulty accurately remembering forensic details about the crime, such as where, when, and who was involved. This could impact the reliability of their statements and testimony in investigations and trials. The novel finding that internally motivated lies are more detrimental to source memory than externally motivated lies implies that witnesses driven by internal desires to lie may struggle more to recall contextual specifics than those pressured to lie externally. This distinction could aid investigators and lawyers in assessing the credibility and accuracy of testimony from suspects lying for self‐gain versus witnesses coerced to lie. Overall, these memory deficiencies associated with deceit highlight the need for caution in evaluating the veracity and precision of witness statements in the justice system. The results underscore the malleability of memory and how lying distorts recollection in ways that can impede due process if not accounted for. Training for legal professionals on using objective evidence to corroborate or expose inconsistencies in testimony may help counteract these effects. Further research on motivation and memory errors in real‐world deceit scenarios is warranted. However, these findings represent an important step in understanding how different motivations for lying differentially affect memory accuracy. Such psychological science insights can inform best practices in the justice system to account for the fallibility of memory in assessing conflicting accounts.

Some limitations in the research methods must be acknowledged. First, the present study utilized both a daily life paradigm and a shopping task as part of its methodology. People shop every day, so shopping is a typical daily life activity. Studying the effect of lying on memory regarding shopping lists may help us obtain a better understanding of the effects of lying on memory in daily life. However, there may be some limitations in the generalizability of these results, as people usually do not lie about their shopping lists. Indeed, people do not generally ask others about their shopping lists in some cultural environments. Moreover, shopping activities are too simple to represent the entire picture of daily life. In reality, an increasing number of daily life activities are more complicated than shopping. Therefore, caution must be applied when generalizing the results. Second, this study does not include an instructed honest group because we have no reason to doubt that memory performance would not differ between instructed and voluntary honesty. However, more work on this issue is needed in the future. Third, there may be some potential limitations when we considered larger *z* or *t* values as indicating greater effects. Without conducting formal statistical tests, we cannot be sure whether the differences in *z* or *t* values are statistically significant or due to chance. Defining “great effects” based on previous studies may not be appropriate when the effects observed in those studies had different sample sizes, designs, and/or methods. Furthermore, participants in the lying groups utilized mixed deceptive strategies, and we did not isolate a specific type of lie. The effect of lying on memory observed in the present study resulted from these mixed lying strategies. Thus, the effect of different deception strategies employed under different deception motives on memory was not fully explored in this study. Future research could address this issue using a more precise and comprehensive design.

In summary, this study applied a daily life paradigm to investigate the effects of lying on memory and whether motivation plays a role in the effects of lying. We found that internally and externally motivated lying can undermine source and destination memories. We also demonstrated that internally motivated lying can exert a greater effect on memory than externally motivated lying. Therefore, internally and externally motivated lies can impair memories, but the former is more disruptive to memories than the latter.

## CONFLICT OF INTEREST STATEMENT

The authors declare that the research was conducted in the absence of any commercial or financial relationships that could be construed as a potential conflict of interest.

## ETHICS STATEMENT

This study was performed in line with the principles of the Declaration of Helsinki. Approval was granted by the Ethics Committee of the School of Education and Psychological Science at Sichuan University of Science and Engineering. Written informed consent was obtained from all individual participants included in the study.
